# Prevalences of sexually transmitted infections in young adults and female sex workers in Peru: a national population-based survey

**DOI:** 10.1016/S1473-3099(12)70144-5

**Published:** 2012-10

**Authors:** César P Cárcamo, Pablo E Campos, Patricia J García, James P Hughes, Geoff P Garnett, King K Holmes, for the Peru PREVEN study team

**Affiliations:** a Epidemiology, STD, and HIV Unit, School of Public Health and Administration, Universidad Peruana Cayetano Heredia, Lima, Peru; b Imperial College, London, London, UK; c Center for AIDS and STD, University of Washington, Seattle, WA, USA; d Department of Global Health, University of Washington, Seattle, WA, USA; e Department of Biostatistics, University of Washington, Seattle, WA, USA; f Department of Medicine, University of Washington, Seattle, WA, USA; g Bill & Melinda Gates Foundation, Seattle, WA, USA

## Abstract

**Background:**

We assessed prevalences of seven sexually transmitted infections (STIs) in Peru, stratified by risk behaviours, to help to define care and prevention priorities.

**Methods:**

In a 2002 household-based survey of the general population, we enrolled randomly selected 18–29-year-old residents of 24 cities with populations greater than 50 000 people. We then surveyed female sex workers (FSWs) in these cities. We gathered data for sexual behaviour; vaginal specimens or urine for nucleic acid amplification tests for *Neisseria gonorrhoeae*, *Chlamydia trachomatis*, and *Trichomonas vaginalis*; and blood for serological tests for syphilis, HIV, and (in subsamples) herpes simplex virus 2 (HSV2) and human T-lymphotropic virus. This study is a registered component of the PREVEN trial, number ISRCTN43722548.

**Findings:**

15 261 individuals from the general population and 4485 FSWs agreed to participate in our survey. Overall prevalence of infection with HSV2, weighted for city size, was 13·5% in men, 13·6% in women, and 60·6% in FSWs (all values in FSWs standardised to age composition of women in the general population). The prevalence of *C trachomatis* infection was 4·2% in men, 6·5% in women, and 16·4% in FSWs; of *T vaginalis* infection was 0·3% in men, 4·9% in women, and 7·9% in FSWs; and of syphilis was 0·5% in men, 0·4% in women, and 0·8% in FSWs. *N gonorrhoeae* infection had a prevalence of 0·1% in men and women, and of 1·6% in FSWs. Prevalence of HIV infection was 0·5% in men and FSWs, and 0·1% in women. Four (0·3%) of 1535 specimens were positive for human T-lymphotropic virus 1. In men, 65·0% of infections with HIV, 71·5% of *N gonorrhoeae*, and 41·4% of HSV2 and 60·9% of cases of syphilis were in the 13·3% who had sex with men or unprotected sex with FSWs in the past year. In women from the general population, 66·7% of infections with HIV and 16·7% of cases of syphilis were accounted for by the 4·4% who had been paid for sex by any of their past three partners.

**Interpretation:**

Defining of high-risk groups could guide targeting of interventions for communicable diseases—including STIs—in the general Peruvian population.

**Funding:**

Wellcome Trust-Burroughs Wellcome Fund Infectious Disease Initiative and US National Institutes of Health.

## Introduction

National population-based surveys of sexually transmitted infections (STIs) and sexual behaviours provide estimates of population-specific prevalences, trends, and determinants.^1^, ^2^ However, most national surveys have been restricted to few STIs and sexual behaviours,^3^, ^4^, ^5^, ^6^, ^7^ or to rural populations.^8^ We present results of surveys of STI prevalences in middle-sized cities in Peru, and of patterns of sexual behaviours in random household samples of young adult residents and samples of female sex workers (FSWs).

## Methods

### Study design and participants

In 2002, we selected 30 Peruvian cities with populations greater than 50 000 people. We excluded Lima because of its size, two other cities because of their proximity to Lima, and three cities involved in a separate prevention study of STIs and HIV—leaving 24 cities for inclusion in our study. The study sampling frame of the general-population survey used the 1999–2000 precensus household database of the Peruvian National Institute of Statistics and Informatics (INEI). We defined sampling units (clusters) as roughly 40 households, providing about ten eligible participants per cluster. For each city, we assigned a random number to every cluster, sorted them numerically, and selected the top 108 clusters (36 each for three fieldworkers). Three of the 2592 neighbourhood clusters randomly selected for this survey were judged to be dangerous (interviewers would be at risk of being attacked or robbed) and not surveyed.

Fieldwork for the general-population survey took place between Aug 15, and Nov 15, 2002, in nine 10-day periods. Fieldworkers updated household lists of men and women aged 18–29 years who had lived in the city for at least the past 6 months in every cluster. In clusters with more than ten households containing young people, a table of random numbers for selection of households was used to obtain a random sample of ten. Four clusters (40 suitable households) were covered per period by each fieldworker. When several household members met our inclusion criteria, the individual with the most recent birthday was selected, and scheduled for interview to confirm eligibility and obtain informed consent. When eligibility was not subsequently confirmed, the eligible person with next most recent birthday was selected. Recruitment continued until the quota of 250 women and 250 men per city was reached or exceeded. Interviewers wore white gowns with INEI and PREVEN logos, and carried backpacks and coolers containing frozen acrylamide gel bags.

Between Nov 7, 2002, and April 22, 2003, local teams used key informants (peer FSWs, FSW clients, and clinics for FSWs) to map sex work venues in every city, such as brothels, bars, discos, hotels, toll booths, massage parlours, parks, and streets. The teams selected the order for venue visits with random numbers until all FSWs identified were recruited, or 200 FSWs had been recruited per city, primarily from these venues (80%) and also from local government clinics serving FSWs (20%). All eligible consenting women aged 14 years or older offering sex services identified during venue or clinic visits were invited to participate. FSW surveyors visited new venues when open for sex services until the city quota of 200 was reached or exceeded, or the survey period ended.

The research team obtained written approval from the Peruvian Ministry of Health, and verbal approval from health directors of all regions surveyed to use Ministry of Health laboratories and counselling staff for the survey. Participants provided verbal informed consent, without personal identifiers. Institutional review boards at the University of Washington (Seattle, WA, USA), the Universidad Peruana Cayetano Heredia (Lima, Peru), and the US Naval Medical Research Unit Number 6 (NAMRU-6; Lima, Peru) approved the protocol, consent forms, and instruments. The institutional review boards approved inclusion of girls aged 14–17 years involved in sex work because they were financially independent from their parents, sexually active, and as minors were often excluded from Ministry of Health clinical services for FSWs. Young adult survey participants received study caps and T-shirts; FSW survey participants received free condoms.

### Procedures

For general-population surveys, female health professionals (ie, nurses, midwives, and medical doctors) with venepuncture experience took a 9-day course of lectures, demonstrations, and supervised role-playing of interviewing, specimen collection and coding, ethics, STIs, map interpretation, participant selection, and informed consent procedures. Seven regional coordinators, a national supervisor, three interviewers, and one supervisor for every city were selected on the basis of course performance. We similarly trained and selected interviewers and supervisors for FSW surveys; every field team included an FSW peer-educator, a counsellor–interviewer, and a physician or midwife.

Laboratory technicians designated by local health authorities received training in specimen processing, labelling, storage, shipping, and *Trichomonas vaginalis* cultures. Staff from the University of Washington provided supervision, quality assurance, and Gen-Probe Aptima Combo 2 assays for quality control.

Face-to-face interviews addressed demographics, household characteristics, travel and migration, labour, and marriage. Subsequent private, self-administered questionnaires of participants from the general population explored sexual behaviours; completed questionnaires were sealed in envelopes, and placed into locked voting bags^9^ to ensure anonymity.

Men from the general population provided 15 mL first-void urine and the women provided self-obtained vaginal swabs (SOVS), with one polyester–plastic swab and then two cotton–wooden swabs. Interviewers promptly placed polyester SOVS into cryovials, and inoculated the first cotton swab into an InPouch TV system (BioMed Diagnostics, White City, OR, USA) for *T vaginalis* culture. Women from the general population who did not provide SOVS were asked for 15 mL first-void urine samples. For FSWs, a polyester cervical swab and two cotton vaginal swabs were obtained during speculum examination. Venous blood was collected from consenting participants. A subset of the general-population participants (100% of individuals with a cohabiting sex partner and 10% random sample of others) and an 8·5% random sample (size of sample determined by number of test kits available) of FSWs underwent testing for herpes simplex virus 2 (HSV2). To obtain the random samples, we assigned random numbers to individuals from the general population and to FSWs who gave blood, ordered them numerically, and selected individuals from the top of the list.

Urine, polyester swabs, and blood were transported inside coolers to the local laboratory within 4 h of collection. *T vaginalis* cultures were transported at ambient temperature. Consent forms, face-to-face questionnaires, referral cards, and samples were labelled with alphanumeric codes and barcodes. Self-completed questionnaires were labelled with bar codes.

Local laboratory staffing was scheduled whenever specimens were obtained; urine and serum were promptly divided into aliquots in vials and frozen at −20°C. Frozen urine and serum aliquots and polyester swabs were sent weekly to Lima laboratories in Styrofoam coolers wrapped in metal. *T vaginalis* culture results were sent to Lima monthly.

Temperature within coolers on arrival in Lima was usually less than 0°C and never more than 1°C. 1 mL of 2-sucrose phosphate medium^10^ was added to vials containing dry polyester swabs, which was then mixed, divided into aliquots, and stored at −20°C until testing by Cobas Amplicor for *Neisseria gonorrhoeae* and *Chlamydia trachomatis*. All specimens positive for *N gonorrhoeae*, samples of specimens positive for *C trachomatis*, and all specimens with equivocal results per manufacturer's instructions went to the University of Washington for confirmatory tests with the Aptima Combo 2 assay. Specimens in 2-sucrose-phosphate also underwent Aptima testing for *T vaginalis*. We tested specimens for syphilis serology with RPR nosticon II (Organon Teknika, Boxtel, Netherlands), judging results positive when rapid plasma reagin measurements were 1:8 or more, with confirmation by Serodia TPPA (Fujirebio, Tokyo, Japan). We used Uni-Form II Ag/Ab (Biomerieux-Vironostika, Durham, NC, USA) ELISA for HIV serology and New LAV Blot I (Bio-Rad, Hercules, CA, USA) for confirmation. The first 1530 sera specimens (number determined by number of test kits available) from the general population were tested for human T-lymphotropic virus seropositivity by ELISA (Vironostika, Durham, NC, USA), with western blot 2·4 (Genelabs Diagnostics, Science Park, Singapore). We used ELISA testing for HSV2 (HerpeSelect HSV-2, Focus Technologies, Cypress, CA, USA); index values of 3·5 or more were deemed positive. All diagnostic tests were done in accordance with the manufacturer's recommended procedures.

Participants who provided specimens received HIV counselling booklets.^11^ Results for syphilis, *C trachomatis*, *N gonorrhoeae*, HIV, and *T vaginalis* culture were printed and distributed to predefined local STI clinics to which participants from the general population had been referred. To distribute results to FSWs, clinic counsellors returned to sex work venues. Results, counselling, and treatment for STIs, and referral for care for patients with HIV (after informed consent) were provided anonymously by matching sample codes with each participant's referral card code.

### Statistical analysis

Face-to-face questionnaires were checked daily for consistency and completeness, corrected locally when necessary, and then sent to Lima for data entry. Self-completed questionnaires were sent to Lima without local validation to ensure confidentiality. We entered general-population data with an SQL program. All FSW survey data were double entered with EpiInfo (version 3.3). All self-completed questionnaires and 5% of face-to-face questionnaires were double entered. We used 5% of face-to-face questionnaires to allow us to test the hypothesis that the frequency of errors in any one variable was less than 2% with 95% CIs. For face-to-face questionnaires, error frequency was less than 1%.

We analysed data with STATA (version 8.2). To account for differences in general-population sampling probabilities in cities of various population sizes, we present weighted prevalences when totalling prevalences of STIs for all cities combined. City-level estimates of general-population prevalences of HSV2 are weighted to account for test subsampling. To account for differences in age composition of FSWs and women from the general population surveyed, we calculated age-standardised STI prevalences for FSWs by excluding those younger than 18 years or older than 29 years, and weighted remaining FSW data in line with proportions of women from the general population in every age category. We calculated prevalence ratios with an unweighted generalised linear model with log link and adjustment for clustering and age. We calculated p values from the same regression or by exact logistic regression for very rare diseases.

This study is a registered component of the PREVEN trial, number ISRCTN43722548.

### Role of the funding source

The sponsors of the study had no role in study design, data collection, data analysis, data interpretation, or writing of the report. The corresponding author had full access to all data in the study and had final responsibility for the decision to submit for publication.

## Results

Of 15 261 individuals from the general population who agreed to participate in our survey (table 1), 43 (0·3%) left the self-completed questionnaire blank. 11 483 individuals (75·2%) provided blood. Of 7486 men, 6500 (86·8%) provided urine. Of 7775 women, 5924 (76·2%) provided SOVS and 588 others (7·6%) provided urine. 4485 FSWs participated. The FSW participation rate was 95% in the five cities where it was measured. Questionnaire data are available from 4413 (98·4%) FSWs, blood from 4428 (98·7%), and cervical swabs (for *C trachomatis* and *N gonorrhoeae*) and vaginal swabs (for *T vaginalis)* from 4280 (95·4%).

**Table 1:** Participation in the general population survey by city

	**Households selected**	Households excluded*	**Selected person absent**	**Selected person identified and invited to participate**				
				**Total**	**Refused**	**Accepted**		
						Total	Provided questionnaire data	Provided biological samples
Arequipa	970	113 (12%)	11	846	82 (10%)	764 (90%)	764	607 (79%)
Ayacucho	969	259 (27%)	13	697	40 (6%)	657 (94%)	657	538 (82%)
Barranca	990	168 (17%)	6	816	114 (14%)	702 (86%)	702	537 (76%)
Cajamarca	995	197 (20%)	3	795	131 (16%)	664 (84%)	664	518 (78%)
Cerro de Pasco	765	106 (14%)	2	657	43 (7%)	614 (93%)	614	566 (92%)
Chimbote	980	261 (27%)	21	698	26 (4%)	672 (96%)	672	564 (84%)
Chincha	925	176 (19%)	0	749	30 (4%)	719 (96%)	719	523 (73%)
Cusco	995	334 (34%)	1	660	56 (8%)	604 (92%)	604	563 (93%)
Huancayo	971	207 (21%)	19	745	67 (9%)	678 (91%)	678	537 (79%)
Huanuco	969	288 (30%)	2	679	61 (9%)	618 (91%)	618	516 (83%)
Huaraz	878	154 (18%)	14	710	76 (11%)	634 (89%)	634	539 (85%)
Ica	866	281 (32%)	1	584	20 (3%)	564 (97%)	564	521 (92%)
Ilo	903	110 (12%)	59	734	152 (21%)	582 (79%)	582	550 (94%)
Iquitos	780	188 (24%)	0	592	11 (2%)	581 (98%)	581	531 (91%)
Juliaca	954	250 (26%)	5	699	91 (13%)	608 (87%)	608	572 (94%)
Pisco	885	242 (27%)	3	640	59 (9%)	581 (91%)	580	546 (94%)
Piura	892	213 (24%)	5	674	33 (5%)	641 (95%)	641	558 (87%)
Pucallpa	993	348 (35%)	3	642	32 (5%)	610 (95%)	610	552 (90%)
Puno	989	234 (24%)	14	741	81 (11%)	660 (89%)	660	583 (88%)
Sullana	984	197 (20%)	12	775	124 (16%)	651 (84%)	651	524 (80%)
Tacna	861	215 (25%)	0	646	48 (7%)	598 (93%)	598	552 (92%)
Talara	967	182 (19%)	29	756	192 (25%)	564 (75%)	564	522 (92%)
Tarapoto	899	236 (26%)	5	658	19 (3%)	639 (97%)	639	544 (85%)
Tumbes	956	282 (29%)	0	674	18 (3%)	656 (97%)	656	536 (82%)
Total	22 336	5241 (23%)	228	16 867	1606 (10%)	15 261 (90%)	15 260	13 099 (86%)

Data are n or n (%).

* Excluded after selection because eligibility of members was not confirmed or because the quota for their sex had already been reached.

Frequencies of STIs varied between cities (Table 2, Table 3, Table 4). In three cities in the Amazon jungle (Tarapoto, Iquitos, and Pucallpa) the prevalence of STIs was notably higher than in other cities, particularly in men (table 2).

**Table 2:** Prevalences of sexually transmitted infections in men aged 18–29 years by region and city

		**Serological test results**			**Nucleic acid amplification testing of urine**		
		Syphilis*	HSV2†	HIV	*Neisseria gonorrhoeae*	*Chlamydia trachomatis*	*Trichomonas vaginalis*
Coastal							
	Barranca	3/213 (1%)	59 (8%)	2/213 (1%)	0/267	10/267 (4%)	0/267
	Chimbote	1/238 (<1%)	47 (9%)	0/238	0/287	8/287 (3%)	0/288
	Chincha	1/231 (<1%)	30 (28%)	2/230 (1%)	1/256 (<1%)	8/256 (3%)	0/256
	Ica	4/232 (2%)	48 (4%)	1/232 (<1%)	0/255	6/256 (2%)	1/256 (<1%)
	Ilo	0/232	62 (2%)	0/231	0/251	10/251 (4%)	1/265 (<1%)
	Pisco	0/259	52 (8%)	0/259	0/265	10/265 (4%)	3/281 (1%)
	Piura	2/234 (<1%)	51 (1%)	0/234	0/278	3/279 (1%)	0/280
	Sullana	0/215	51 (10%)	1/215 (1%)	0/242	11/243 (5%)	0/268
	Tacna	0/243	36 (5%)	1/243 (<1%)	0/244	10/244 (4%)	1/264 (<1%)
	Talara	1/223 (<1%)	45 (18%)	1/224 (<1%)	1/255 (<1%)	12/255 (5%)	1/255 (<1%)
	Tumbes	0/225	45 (22%)	1/225 (<1%)	1/265 (<1%)	5/265 (2%)	0/265
Andean							
	Arequipa	0/213	26 (9%)	1/213 (1%)	0/273	5/273 (2%)	0/275
	Ayacucho	0/259	51 (8%)	0/259	0/270	11/270 (4%)	1/272 (<1%)
	Cajamarca	0/219	61 (5%)	0/219	0/231	7/231 (3%)	0/258
	Cerro de Pasco	0/252	45 (1%)	1/252 (<1%)	0/253	14/253 (6%)	0/280
	Cuzco	1/252 (<1%)	60 (19%)	0/252	0/284	12/285 (4%)	2/286 (1%)
	Huancayo	0/219	40 (5%)	0/219	0/255	11/255 (4%)	2/256 (1%)
	Huaraz	0/225	42 (7%)	0/225	0/265	13/265 (5%)	1/265 (<1%)
	Juliaca	0/261	50 (0%)	0/261	1/244 (<1%)	12/244 (5%)	2/289 (1%)
	Puno	0/265	60 (<1%)	2/265 (1%)	0/247	14/247 (6%)	2/300 (1%)
Amazonian							
	Huanuco	0/210	59 (9%)	0/210	2/234 (1%)	13/235 (6%)	4/251 (2%)
	Iquitos	4/252 (2%)	51 (38%)	4/252 (2%)	0/267	21/267 (8%)	1/268 (<1%)
	Pucallpa	5/260 (2%)	55 (34%)	5/260 (%2)	1/286 (<1%)	15/286 (5%)	0/286
	Tarapoto	6/250 (2%)	50 (24%)	0/250	2/258 (1%)	8/258 (3%)	0/267
Total		28/5682 (1%)	1176	22/5681 (<1%)	9/6232 (<1%)	249/6237 (4%)	22/6498 (<1%)
Weighted prevalence‡		0·5%	13·5%	0·5%	0·1%	4·2%	0·3%

Data are number positive/number tested (%) unless otherwise stated. HSV2=herpes simplex virus 2.

* Rapid plasma reagin 1:8 or higher, *Treponema pallidum* particle agglutination positive.

† HSV2 testing was done with a subset of sera available, and thus city prevalences of seropositivity are further weighted accordingly and only denominators are shown.

‡ Prevalences weighted by city size.

**Table 3:** Prevalences of sexually transmitted infections in women aged 18–29 years from the general population by region and city

		**Serological test results**			**Nucleic acid amplification testing of urine or vaginal swab**		
		Syphilis*	HSV2†	HIV	*Neisseria gonorrhoeae*	*Chlamydia trachomatis*	*Trichomonas vaginalis*
Coastal							
	Barranca	0/230	55 (4%)	0/230	1/268 (<1%)	17/267 (6%)	14/263 (5%)
	Chimbote	0/237	52 (16%)	0/237	0/271	12/271 (4%)	13/270 (5%)
	Chincha	2/246 (1%)	63 (22%)	0/247	0/263	17/263 (7%)	21/262 (8%)
	Ica	1/243 (<1%)	49 (19%)	0/243	0/262	8/261 (3%)	12/262 (5%)
	Ilo	0/254	68 (6%)	0/254	0/276	15/276 (5%)	6/276 (2%)
	Pisco	0/228	61 (9%)	1/228 (<1%)	0/258	17/259 (7%)	24/260 (9%)
	Piura	0/243	68 (3%)	0/243	0/274	12/274 (4%)	12/273 (4%)
	Sullana	0/218	64 (14%)	0/218	0/249	9/249 (4%)	6/254 (2%)
	Tacna	2/260 (1%)	51 (14%)	0/260	1/283 (<1%)	22/283 (8%)	11/283 (4%)
	Talara	1/242 (<1%)	67 (10%)	0/242	1/263 (<1%)	15/264 (6%)	17/261 (7%)
	Tumbes	0/242	72 (17%)	0/242	1/266 (<1%)	9/268 (3%)	6/268 (2%)
Andean							
	Arequipa	0/205	48 (9%)	0/204	0/296	12/296 (4%)	6/298 (2%)
	Ayacucho	0/253	60 (9%)	0/253	0/265	19/264 (7%)	16/262 (6%)
	Cajamarca	0/219	51 (2%)	0/219	0/250	15/250 (6%)	17/254 (7%)
	Cerro de Pasco	0/270	64 (12%)	0/270	0/282	14/283 (5%)	24/285 (8%)
	Cuzco	2/244 (1%)	69 (2%)	0/244	0/272	16/272 (6%)	15/272 (6%)
	Huancayo	1/244 (<1%)	49 (9%)	0/244	0/272	24/273 (9%)	18/272 (7%)
	Huaraz	0/247	61 (5%)	0/247	1/272 (<1%)	19/272 (7%)	18/270 (7%)
	Juliaca	0/237	79 (1%)	0/237	0/268	26/268 (10%)	16/272 (6%)
	Puno	0/235	64 (5%)	0/235	1/278 (<1%)	21/278 (8%)	9/282 (3%)
Amazonian							
	Huanuco	2/238 (1%)	47 (8%)	1/238 (<1%)	0/263	16/262 (6%)	18/263 (7%)
	Iquitos	4/255 (2%)	66 (36%)	0/255	0/257	26/258 (10%)	14/260 (5%)
	Pucallpa	2/245 (1%)	67 (33%)	1/245 (<1%)	0/258	29/258 (11%)	12/260 (5%)
	Tarapoto	3/264 (1%)	91 (17%)	0/264	1/273 (<1%)	24/273 (9%)	11/275 (4%)
Total		20/5799 (<1%)	1486	3/5799 (<1%)	7/6439 (<1%)	414/6442 (6%)	336/6457 (5%)
Weighted prevalence‡		0·4%	13·6%	0·1%	0·1%	6·5%	4·9%

Data are number positive/number tested (%) unless otherwise stated. HSV2=herpes simplex virus 2.

* Rapid plasma reagin 1:8 or higher, *Treponema pallidum* particle agglutination positive.

† HSV2 testing was done with a subset of sera available, and thus city prevalences of seropositivity are further weighted accordingly and only denominators are shown.

‡ Prevalences weighted by city size.

**Table 4:** Prevalences of sexually transmitted infections in female sex workers by region and city

		**Serological test results**			**Nucleic acid amplification testing of vaginal swabs**		
		Syphilis*	HSV2†	HIV	*Neisseria gonorrhoeae*	*Chlamydia trachomatis*	*Trichomonas vaginalis*
Coastal							
	Barranca	3/170 (2%)	14/18 (78%)	0/168	4/141 (3%)	12/141 (9%)	8/136 (5%)
	Chimbote	6/197 (3%)	32/36 (89%)	2/199 (1%)	1/200 (1%)	21/200 (11%)	14/192 (7%)
	Chincha	0/196	5/6 (83%)	3/199 (2%)	0/200	8/200 (4%)	11/186 (6%)
	Ica	0/198	6/9 (67%)	1/200 (1%)	0/198	11/198 (6%)	9/184 (5%)
	Ilo	0/200	5/11 (45%)	0/200	2/200 (1%)	32/200 (16%)	8/196 (4%)
	Pisco	3/148 (2%)	3/7 (43%)	1/148 (1%)	0/148	16/148 (11%)	17/145 (12%)
	Piura	2/193 (1%)	8/11 (73%)	4/193 (2%)	3/171 (2%)	17/171 (10%)	14/161 (9%)
	Sullana	2/197 (1%)	14/27 (52%)	0/200	1/197 (1%)	24/197 (12%)	22/192 (11%)
	Tacna	0/172	6/10 (60%)	1/205 (1%)	0/196	39/196 (20%)	15/193 (8%)
	Talara	0/152	5/12 (42%)	2/143 (1%)	0/156	21/156 (13%)	12/155 (8%)
	Tumbes	2/72 (3%)	10/12 (83%)	2/74 (3%)	1/57 (2%)	6/57 (11%)	3/53 (6%)
Andean							
	Arequipa	1/198 (1%)	4/10 (40%)	1/201 (1%)	1/188 (1%)	42/188 (22%)	21/194 (11%)
	Ayacucho	2/143 (1%)	9/15 (60%)	1/147 (1%)	1/147 (1%)	18/147 (12%)	8/143 (6%)
	Cajamarca	2/177 (1%)	9/12 (75%)	0/184	0/196	32/196 (16%)	21/184 (11%)
	Cerro de Pasco	1/194 (1%)	3/17 (18%)	0/199	0/200	40/200 (20%)	24/196 (12%)
	Cusco	1/208 (1%)	10/17 (59%)	0/208	0/200	21/200 (11%)	18/194 (9%)
	Huancayo	1/194 (1%)	5/10 (50%)	0/196	2/185 (1%)	24/185 (13%)	7/181 (4%)
	Huaraz	1/143 (1%)	8/11 (73%)	1/140 (1%)	1/138 (1%)	22/138 (16%)	12/137 (9%)
	Juliaca	1/194 (1%)	1/11 (9%)	0/197	2/177 (1%)	29/177 (16%)	8/171 (5%)
	Puno	0/200	4/14 (29%)	0/201	3/183 (2%)	32/183 (17%)	13/182 (7%)
Amazonian							
	Huanuco	7/203 (3%)	16/21 (76%)	1/202 (1%)	7/204 (3%)	46/204 (23%)	40/194 (21%)
	Iquitos	9/197 (5%)	26/26 (100%)	3/200 (2%)	20/198 (10%)	36/198 (18%)	17/175 (10%)
	Pucallpa	7/195 (4%)	31/32 (97%)	3/200 (2%)	8/181 (4%)	36/181 (20%)	17/171 (10%)
	Tarapoto	5/173 (3%)	23/26 (89%)	3/159 (2%)	12/202 (6%)	48/202 (24%)	24/190 (13%)
Total		56/4314 (1%)	257/381 (67%)	29/4363 (1%)	69/4263 (2%)	633/4263 (15%)	363/4105 (9%)
Age-adjusted prevalence‡		0·8%	60·6%	0·5%	1·6%	16·4%	7·9%

Data are number positive/number tested (%) unless otherwise stated. HSV2=herpes simplex virus 2.

* Rapid plasma reagin 1:8 or higher, *Treponema pallidum* particle agglutination positive.

† HSV2 testing done with a self-weighted subset of sera available.

‡ Restricted to 18–29-year-old female sex workers, and standardised to the age composition of women in the general populations survey (age compositions in the two groups were very similar).

6069 men (81·1%) and 5623 women (72·3%) reported previous sexual intercourse. Prevalences were generally lower in participants denying sexual experience than in those reporting previous sexual intercourse (table 5). Only four (0·3%) of 1530 sera specimens from individuals from the general population were positive for human T-lymphotropic virus 1 (two men, two women), and none were positive for human T-lymphotropic virus 2.

**Table 5:** Prevalences of infections

	**Men**		**Women**	
	Sexually experienced (n=6069)	Not sexually experienced (n=1417)	Sexually experienced (n=5623)	Not sexually experienced (n=2152)
Herpes simplex virus 2*	14·6%	6·6%	17·4%	1·3%
HIV	0·4%	0·2%	0·1%	0
Syphilis†	0·5%	0·5%	0·4%	0·2%
*Chlamydia trachomatis*	4·6%	1·2%	7·8%	2·0%
*Trichomonas vaginalis*	0·4%	0	5·8%	3·3%
*Neisseria gonorrhoeae*	0·1%	0·2%	0·1%	0·1%

* Weighted values.

† Rapid plasma reagin 1:8 or higher, *Treponema pallidum* particle agglutination positive.

Of sexually experienced men from the general population, 460 (7·6%) reported sex with another man and 447 (7·4%) unprotected sex with an FSW within the past year. Of 5607 sexually experienced women from the general population for whom responses to the three relevant questions were available, 233 (4·2%) reported receiving money in exchange for sex from any of their past three sex partners. In most risk groups of men and women from the general population and in FSWs, HSV2 seropositivity had the highest prevalence, then *C trachomatis* infection, and then *T vaginalis* infection in women (figure, table 6). Prevalences of the various STIs clearly differ by risk group (table 6).

**Figure:**
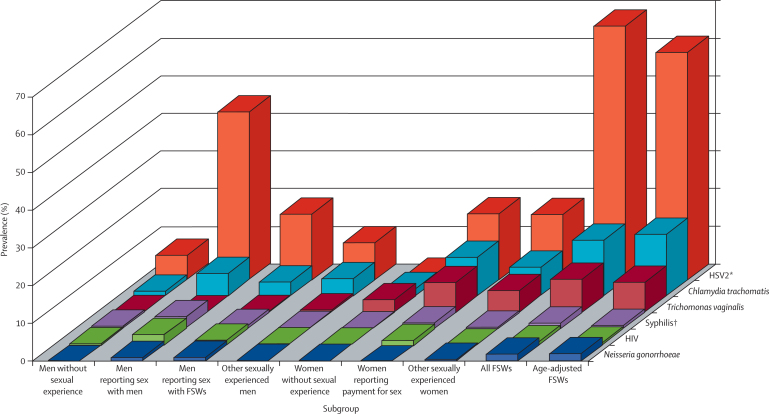
Prevalences of sexually transmitted infections by subgroups of men and women aged 18–29 years from the general population, in all FSWs, and in age-adjusted FSWs Age-adjusted data for FSWs aged 18–29 years were weighted for comparison with women from the general population. FSW=female sex worker. HSV2=herpes simplex virus 2. *HSV2 testing was done with a subset of sera available, and thus city prevalences of seropositivity are further weighted accordingly. †Rapid plasma reagin 1:8 or higher, *Treponema pallidum* particle agglutination positive.

**Table 6:** Prevalences of sexually transmitted infections by risk category

	**Men**				**Women**				
	Without sexual experience (n=1129)	Had sex with men in past year (n=432)	Had unprotected sex with FSW in past year (n=284)*	Others with sexual experience (n=4689)	From general population without sexual experience (n=1512)	From general population and paid for sex by any of last three partners (n=219)	Others from general population with sexual experience (n=4792)	All FSWs (n=4463)	Age-adjusted sample of FSWs (n=4463)†
*Neisseria gonorrhoeae*	0·19%	0·73%	0·73%	0·04%	0·07%	0·00%	0·13%	1·62%	1·64%
HIV	0·22%	2·52%	1·13%	0·17%	0·00%	1·04%	0·02%	0·66%	0·52%
Syphilis‡	0·54%	3·02%	0·75%	0·22%	0·16%	1·55%	0·35%	1·30%	0·78%
*Trichomonas vaginalis*	0	0·00%	0·36%	0·45%	3·29%	7·83%	5·70%	8·84%	7·87%
*Chlamydia trachomatis*	1·21%	6·02%	3·64%	4·49%	1·89%	10·23%	7·68%	14·85%	16·41%
HSV2§	6·63%	44·66%	17·47%	10·00%	1·29%	17·60%	17·42%	67·45%	60·58%

Patients in each risk category are those who provided questionnaire data and had at least one laboratory test result. FSW=female sex worker. HSV2=herpes simplex virus 2.

* Excludes individuals who also had sex with men.

† 18–29-year-old FSWs, standardised to the age composition of women in the general-population survey.

‡ Rapid plasma reagin 1:8 or higher, *Treponema pallidum* particle agglutination positive.

§ HSV2 seroprevalence is weighted by city size and sampling probability.

Additionally, STI prevalence ratios showed differences between risk subgroups (appendix). Compared with sexually experienced women from the general population not receiving money for sex, FSWs had significantly higher age-adjusted prevalences of *N gonorrhoeae* (prevalence ratio 12·31, p=0·004), HSV2 (3·12, p<0·001), syphilis (3·04, p<0·001), *C trachomatis* (2·09, p<0·001), and *T vaginalis* (1·56, p<0·001; appendix). Furthermore, prevalence of syphilis in women from the general population receiving money for sex from any of the past three partners was significantly higher (4·77, p=0·016) and prevalence of HIV was also increased (40·17, p=0·010; appendix) compared with sexually experienced women who did not receive money for sex. Women denying sexual experience had lower prevalences of all STIs than did sexually experienced women, although the difference was significant only for infection with *C trachomatis* (0·19, p<0·001), HSV2 (0·25, p=0·007), and *T vaginalis* (0·57, p<0·001; appendix).

Compared with sexually experienced men reporting neither sex with men nor unprotected sex with FSWs during the past year, men reporting sex with men had significantly higher prevalences of HIV, syphilis, *N gonorrhoeae*, and HSV2 (appendix). Exclusively heterosexual men reporting unprotected sex with FSWs during the past year had significantly higher prevalences of *N gonorrhoeae* and HIV, and men denying sexual experience had significantly lower prevalences of *C trachomatis* and *T vaginalis* infection than did sexually experienced individuals not reporting unprotected sex with FSWs (appendix). In men and women from the general population denying sexual experience, HSV2 seropositivity, *C trachomatis*, and (in women only) *T vaginalis*, were nonetheless occasionally reported (appendix).

Of 5405 sexually experienced men from the general population for whom laboratory results were available, 432 (8·0%) reported sex with men and 284 (5·3%) exclusively heterosexual men reported unprotected sex with FSWs in the past year. These men accounted for 65·0% of infections with HIV, 60·9% of cases of syphilis, 71·5% of infections with *N gonorrhoeae*, and 41·4% of infections with HSV2, but only 14·8% of *C trachomatis* and 4·5% of *T vaginalis* infections. Of 5011 sexually experienced women from the general population for whom laboratory results were available, 219 (4·4%) reported sex for money with any of their past three partners. These women accounted for 66·7% of infections with HIV and 16·7% of cases of syphilis, but only 0·1% of *N gonorrhoeae,* 5·1% of HSV2, 5·7% of *C trachomatis*, and 5·9% of *T vaginalis* infections.

## Discussion

We have shown that, even in household samples, women reporting receiving money for sex accounted for most infections with HIV in women from the general population. Men who have sex with men and heterosexual men reporting unprotected sex with FSWs accounted for most HIV and *N gonorrhoeae* infections and cases of syphilis in men from the general population. Differences between the sexes in the general population for syphilis and HIV infection were largely a result of concentration of these diseases in men who have sex with men. Survey participation rates were high; detailed sociobehavioral and STI test data allowed us to definitively show that STI prevalences differ greatly between risk groups. These data, when coupled with large numbers of sexual partners for individuals in high-risk groups, clearly suggest that targeted, strong preventive interventions are needed in the general population and for FSWs. The results provided baseline data for an urban community-randomised trial of STI prevention—the Peru PREVEN study.^12^

Investigators of Latin American studies in 1991 and 1992 (of 18–30-year-old patients undergoing routine health examinations in Lima, Peru, and 17–30-year-old sex workers in Mexico City, Mexico) recorded HSV2 seroprevalences^13^ that are similar to the values reported by us for young adults and FSWs in Peru. Infections with *N gonorrhoeae* and HIV and cases of syphilis were absent or nearly absent in many Peruvian cities, even in FSWs. Peru's approach to control of these STIs, at least in heterosexual individuals, warrant continuation. The high prevalences of *C trachomatis* and *T vaginalis* call for innovative improvements in control of both infections. Even higher prevalences of syphilis, *C trachomatis*, and (for women) of *T vaginalis* than in our study have been reported in samples of socially marginalised men (eg, gang members) and women in three coastal cities of Peru,^14^, ^15^, ^16^ suggesting that preventive interventions for such populations will also be important, although challenging to implement. Reasons for substantially higher rates of STIs in certain cities—particularly Amazonian cities—will be further explored in community-level analyses.

The PREVEN surveys extend comprehensive surveillance results from US studies (panel) to Latin America. PREVEN complements the US studies because of the high rates of participation and completion of questionnaires; the assessment of prevalence not only by age, sex, and (in men) sexual orientation, but also by region and history of sex for money; and the surveying of FSWs.

Panel **Research in context**

**Systematic review**

We used PubMed to search Medline with the term “population based survey prevalence” in combination with each pathogen we studied. We identified reports of surveys temporally overlapping with the period 2000–03 published in English in which participants were specifically tested for several sexually transmitted infections (STIs), including the National Health and Nutrition Exam Survey (NHANES) and National Longitudinal Study of Adolescent Health (Add Health) in the USA. We did not include several other population-based surveys that focused on only *Chlamydia trachomatis*, HIV infection, or history of STIs. Population-based data for STIs other than *C trachomatis* or HIV infections were scarce, except in the USA and Peru. During the time of our surveys, NHANES and Add Health examined prevalences of several STIs in young adults in the USA. In 5767 individuals aged 18–49 years in the 2001–04 NHANES, syphilis seroprevalence (rapid plasma reagin titre 1:8 or more) was 0·08%.^17^ The 1999–2004 NHANES used immunodot assays for herpes simplex virus 2 (HSV2) in 2412 adults aged 20–29 years, and showed that prevalence of HSV2 seropositivity in men was 5·6% and in women 15·6%.^18^ In the 2001–04 NHANES, prevalence of *Trichomonas vaginalis* in urine by PCR was 2·2% in 20–29-year-old women.^3^ In the 1999–2002 NHANES,^19^ ligase chain reaction tests of urine showed that prevalence of *Neisseria gonorrhoeae* was 0·21% in men and women combined, and prevalence of *C trachomatis* was 1·9% in women and 3·2% in men. Similarly, in wave 3 (2001–02) of Add Health—a sample of 14 322 18–26-year-old adults—prevalence of *C trachomatis* by ligase chain reaction was 4·7% in women and 3·1% in men, and prevalence of *N gonorrhoeae* was 0·42% in women and 0·44% in men.^20^ Prevalence of *T vaginalis* was 2·8% in women and 1·7% in men.^21^ HIV prevalence measured with oral mucosal transudate assay was 0·1% in men and 0·09% in women, but only 0·02% in individuals not classified as non-Hispanic black.^22^ A national stratified probability sample of 3426 individuals aged 20–64 years surveyed in 1999–2000 in China with urine ligase chain reaction^23^ established that prevalence of *N gonorrhoeae* was 0·08% in women and 0·02% in men. Prevalence of *C trachomatis* was 2·7% in women and 3·3% in men aged 20–34 years. The Natsal 2000 stratified probability survey in Britain (1999–2001) showed that prevalence of *C trachomatis* measured with urine ligase chain reaction was 3·0% in men 25–34-years-old, and 3·0% in women aged 18–24 years.^24^ Finally, in 2011, WHO estimated prevalences of STIs with 2002–05 data from South and Central America and the Caribbean for 15–49-year-old women and men.^25^

**Interpretation**

We have reported prevalences of STIs that are generally higher than are those that have been recorded previously. However, the new prevalence estimates reported by WHO are generally similar to what we have reported, although frequency of *T vaginalis* and *N gonorrhoeae* infection was lower in our study than the WHO estimates. Our results for subgroups and those from NHANES and Add Health, showing that a small number of high risk groups account for most cases of STIs, could guide targeting of interventions in the future in similar settings. Additionally, our finding that STIs are more common in Amazonian cities indicates that preventive interventions for individuals who live in these regions are important.

We excluded Lima, which has had increased rates of HIV infection in men who have sex with men^26^ and post-partum women;^27^ we also excluded rural populations. PREVEN study leaders have previously surveyed STIs in rural women throughout Peru.^28^ Our general-population survey cannot be generalised to populations that are younger or older than ours was, to non-household-based populations, or to individuals living in a city for less than 6 months. Exclusion of non-household populations has been argued to have little effect on national HIV seroprevalence estimates for household surveys;^29^ household-based HIV prevalence surveys could provide useful incidence estimates.^30^

Overall, national surveys can inform national health care and prevention policies, and could motivate similar surveillance elsewhere. Global burden of disease analyses rely on such data. Definition of risk factors in such surveys, and concurrent surveys from high-risk populations, will be increasingly important for targeting of interventions for communicable diseases.
