# Supplementary material for: Prevalences of sexually transmitted infections in young adults and female sex workers in Peru: a national population-based survey
Source: Lancet Infect Dis. 2012 Oct;12(10):765–73. doi: 10.1016/S1473-3099(12)70144-5 (PMC3459082; doi:10.1016/S1473-3099(12)70144-5)
Supplement: Supplementary appendix [file mmc1.pdf]

THE LANCET Infectious Diseases

Supplementary webappendix

This webappendix formed part of the original submission and has been peer reviewed. We post it as supplied by the authors.

Supplement to: Cárcamo CP, Campos PE, García PJ, Hughes JP, Garnett GP, Holmes KK, for the Peru PREVEN study team. Prevalences of sexually transmitted infections in young adults and female sex workers in Peru: a national population-based survey. *Lancet Infect Dis* 2012; published online Aug 7. http://dx.doi.org/10.1016/S1473-3099(12)70144-5.

Appendix

Prevalence ratios for the association between risk groups* and STI among women, adjusted for age and clustering

	NG		HIV		Syphilis≥1:8		TV		CT		HSV-2	
	PR	p+	PR	p+	PR	p	PR	p	PR	p	PR	p
Other SE women, GPS	1		1		1		1		1		1	
FSW, GPS	0.00	0.999	40.17	0.010	4.77	0.016	1.38	0.115	1.33	0.249	0.94	0.839
FSW survey	12.31	0.004	26.02	0.003	3.04	<0.001	1.56	<0.001	2.09	<0.001	3.12	<0.001
Non-SE women	0.39	0.468	0.00	1.000	0.59	0.518	0.57	<0.001	0.19	<0.001	0.25	0.007

* Risk categories for men include non-sexually experienced (non-SE) men, men reporting sex with other men during the past year, clients reporting unprotected sex with FSW during the past year, and other SE men. Risk categories for women from the GP include non-SE women, women reporting sex for money with any of the last three partners, and other SE women.

\+ Due to small numbers of cases, these p-values are based on exact logistic regression without adjustment for clustering.

Prevalence ratios for the association between risk groups* and STI among men, adjusted for age and clustering

	NG		HIV		Syphilis≥1:8		TV		CT		HSV-2	
	PR	p+	PR	p	PR	p	PR	p+	PR	p	PR	p
Other SE men, GPS	1		1		1		1		1		1	
MSM, GPS	14.97	0.014	27.36	<0.001	15.80	<0.001	0.00	0.311	1.36	0.077	2.30	<0.001
Clients, GPS	14.58	0.036	11.52	0.011	2.52	0.264	0.79	1.000	0.83	0.674	1.18	0.621
Non-SE men	3.59	0.514	2.02	0.282	3.08	0.134	0.00	0.028	0.27	<0.001	1.30	0.515

* Risk categories for men include non-sexually experienced (non-SE) men, men reporting sex with other men during the past year, clients reporting unprotected sex with FSW during the past year, and other SE men. Risk categories for women from the GP include non-SE women, women reporting sex for money with any of the last three partners, and other SE women.

\+ Due to small numbers of cases, these p-values are based on exact logistic regression without adjustment for clustering.
